# The relationship between aberrant methylation and survival in non-small-cell lung cancers

**DOI:** 10.1038/sj.bjc.6602013

**Published:** 2004-07-20

**Authors:** S Toyooka, M Suzuki, R Maruyama, K O Toyooka, K Tsukuda, Y Fukuyama, T Iizasa, M Aoe, H Date, T Fujisawa, N Shimizu, A F Gazdar

**Affiliations:** 1 Hamon Center for Therapeutic Oncology Research, University of Texas Southwestern Medical Center, Dallas TX, USA; 2 Department of Cancer and Thoracic Surgery, Okayama University Medical School, Okayama, Japan; 3 Department of Surgery 2, Kyushu University Faculty of Medicine, Fukuoka Japan; 4 Department of Pathology, University of Texas Southwestern Medical Center, Dallas TX, USA

**Keywords:** methylation, *p16*^*INK4a*^, non-small-cell lung cancer, adenocarcinoma of lung

## Abstract

The present study examined the relationship between methylation of five genes (*p16*^*INK4a*^, *RASSF1A*, *APC*, *RARβ* and *CDH13*) and patient survival in 351 cases of surgically resected lung cancers. While there was no relationship between the other genes and survival, *p16*^*INK4a*^ methylation was significantly related to unfavourable prognosis in lung adenocarcinomas.

Lung cancer is the leading cause of cancer deaths in the world. Human lung cancers are classified into two major histologic types, small-cell lung cancer (SCLC) and non-small-cell lung cancer (NSCLC), the latter consisting of several subtypes. Previously, squamous cell carcinoma was the predominant form of NSCLC, but in the last few decades it has been replaced by adenocarcinoma. Moreover, adenocarcinoma is the most common type of lung cancer in women, never smokers and young subjects.

Aberrant methylation of CpG islands in the promoter region of tumour suppressor genes (TSGs) and tumour-related genes has become established as the major mechanism for gene silencing (Baylin *et al*, 1998). Inactivation of TSGs by DNA methylation is regarded as one of the fundamental processes for the development of human malignant tumours, including lung cancers (Zochbauer-Muller *et al*, 2001). We studied the methylation status of five genes, *p16*^*INK4a*^, *RASSF1A*, *APC*, *RARβ* and *CDH13,* that are frequently methylated in lung cancers and that are considered to play an important role in the molecular pathogenesis of lung cancers (Toyooka *et al*, 2001b). We previously reported that smoke exposure, histologic type and geography-related differences in the methylation profiles of NSCLC (Toyooka *et al*, 2003). Some reports have described that methylation of specific TSGs are negative prognostic factors for lung cancer (Tang *et al*, 2000; Burbee *et al*, 2001). In this study, we collected the survival data of 351 cases of NSCLCs and correlated the methylation status of five genes (*p16*^*INK4a*^, *RASSF1A*, *APC*, *RARβ* and *CDH13*) with clinicopathological factors to investigate the effect of methylation of five genes on the survival of patients undergoing curative intent surgical resections for lung cancer.

## MATERIALS AND METHODS

We studied frozen specimens of 351 tumours stored at −80°C obtained from Japanese patients with primary NSCLCs treated by curative intent surgical resection between 1993 and 2000 in our institutions. The patients consisted of 234 males and 117 females and their median age was 65 years. Most of the tumours (325, 93%) were adenocarcinomas (199, 57%) or squamous cell carcinomas (126, 36%), while the remainder consisted of 19 large-cell carcinomas, six adenosquamous cell carcinomas and one unclassified type. In all, 169 patients had stage I disease, 60 stage II, 122 stage III. A total of 252 patients (72%) were ever smokers with a median smoke exposure of 45.9 pack years and 99 never smokers. Institutional Review Board permission and informed consent were obtained at each collection site.

Genomic DNA was isolated from frozen tumour tissue by SDS/proteinase K (Life Technologies Inc., Rockville, MD, USA) digestion, phenol–chloroform extraction and ethanol precipitation. The methylation status of five genes reported to be frequently methylated and silenced in tumour but not in nonmalignant lung tissues (*p16*^*INK4a*^, *RASSF1A*, *APC, RARβ* and *CDH13*) (Toyooka *et al*, 2003, 2004) was determined by methylation-specific PCR (polymerase chain reaction) (MSP) assay using gene-specific primers (Herman *et al*, 1996; Cote *et al*, 1998; Tsuchiya *et al*, 2000; Burbee *et al*, 2001; Toyooka *et al*, 2001a). Briefly, 1 *μ*g of genomic DNA was modified by sodium bisulphite, which converts all unmethylated cytosines to uracils residues while methylated cytosines remain unchanged. Polymerase chain reaction amplification was performed with sodium bisulphate-treated DNA as template as described previously (Herman *et al*, 1996). The MSP assays were sensitive enough to detect one methylated allele in the presence of 10^3^–10^4^ unmethylated alleles (Toyooka *et al*, 2003). DNA from peripheral blood lymphocytes and buccal mucosa brushes, each from 10 of nonsmoking healthy subjects, along with water blanks were used as negative controls for the methylated genes. DNA from lymphocytes healthy volunteer artificially methylated by treatment with Sss1 (New England BioLabs, Beverly, MA, USA) and then subjected to bisulphite treatment was used as a positive control for methylated alleles. Polymerase chain reaction products were visualised on 2% agarose gels stained with ethidium bromide. Results were confirmed by repeat MSP assays after an independently performed bisulphite treatment.

The overall survival was calculated from the date of surgery until the date of death or the last follow-up. Univariate analysis of overall survival was carried out by the Kaplan–Meier method using the log-rank test. Multivariate overall survival analysis was carried out by Cox's proportional-hazards model. The stepwise procedure was used to select independent variables with backward elimination method with *P*-values of entry of 0.10 and rejection of 0.12. All data were analysed with StatView for Windows (SAS Institute Inc., Cary, NC, USA).

## RESULTS

The rates of methylation of five genes were determined in 351 cases of NSCLCs. Aberrant methylation was detected in 86 (25%) of 351 cases for *p16*^*INK4a*^, 120 cases (34%) for *RASSF1A*, 131 cases (37%) for *APC*, 98 cases (28%) for *RARβ* and 104 cases (30%) for *CDH13*. We correlated the methylation status of five genes and clinicopathological factors including gender, age, smoking status, histological differentiation and TNM stage with prognosis for patient. The analysis was performed on all 351 cases, and the major histological subtypes adenocarcinomas and squamous cell carcinomas. As adenocarcinomas are the predominant form of lung cancer in never smokers, the percentage of adenocarcinoma patients who smoked (55%) is lower than the figure for the overall patient population (72%).

With regard to patient prognosis, there was no gene that was correlated with survival in all cases or squamous cell carcinomas. However, methylation of only one gene, *p16*^*INK4a*^, was associated with poor survival by Kaplan–Meier survival analysis (*P*=0.020) in adenocarcinoma (Table 1

**Table 1:** Univariate and multivariate overall survival analysis (a) of all cases of lung adenocarcinomas and (b) 105 cases of stage I lung adenocarcinomas

			***P*-value**	
**Variable**	**Subset**	**No.**	**Univariate^a^**	**Multivariate^b^**
*(a)*				
Gender	Male	104	0.092	NS
	Female	95		
Age (years)	<65	115	0.15	0.069
	65>	84		(RR 1.46; 95% CI, 0.98–2.21)
Smoking status	Ever smoker	110	0.11	NS
	Never smoker	89		
Differentiation	Poor	41	0.15	NS
	Moderate	98		
	Well	60		
T stage	T3 and 4	45	0.0002	0.006
	T1 and 2	154		(RR 1.87; 95% CI, 1.20–2.92
*N*	+	78	<0.0001	<0.0001
	−	121		(RR 4.60; 95% CI, 3.00–7.05)
Stage	I and II	130	< 0.0001	NS
	III	69		
*p16*^*INK4a*^ (M)	+	33	0.020	0.019
	−	166		(RR 1.82; 95% CI, 1.10–3.00)
*RASSF1A* (M)	+	69	0.16	NS
	−	130		
*APC* (M)	+	88	0.33	NS
	−	111		
*RARβ* (M)	+	65	0.28	NS
	−	134		
*CDH13* (M)	+	63	0.26	NS
	−	136		
				
*(b)*				
Gender	Male	47	0.60	NS
	Female	58		
Age (years)	<65	59	0.08	NS
	65>	46		
Smoking status	Smoker	52	0.33	NS
	Never smoker	53		
Differentiation	Poor	20	0.58	NS
	Moderate	43		
	Well	42		
T stage	T1	64	0.19	NS
	T2	41		
*p16*^*INK4a*^ (M)	+	18	0.022	0.022
	−	87		(RR 2.57; 95% CI, 1.15–5.76)
*RASSF1A* (M)	+	33	0.22	NS
	−	72		
*APC* (M)	+	46	0.091	NS
	−	59		
*RARβ* (M)	+	29	0.92	NS
	−	76		
*CDH13* (M)	+	34	0.72	NS
	−	71		

M=methylated; RR=risk ratio; CI=confidence interval; NS=not selected in stepwise Cox's proportional regression model.

a Analysis with log-rank test.

b Analysis with stepwise Cox's proportional-hazards regression model.

a and Figure 1

**Figure 1:**
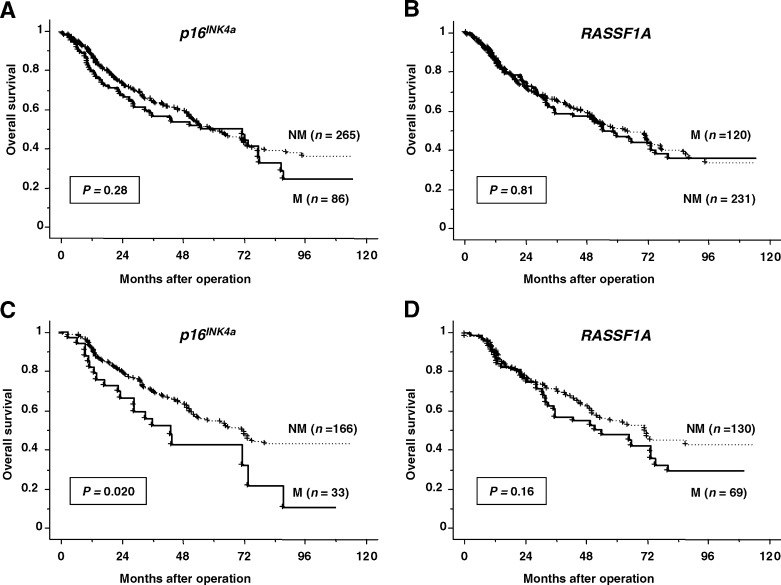
Relationship between methylation status and patient survival by Kaplan–Meier method. (**A**) Survival curves by methylation status of *p16*^*INK4a*^ in all cases. (**B**) Survival curves by methylation status of *RASSF1A* in all cases. (**C**) Survival curves by methylation status of *p16*^*INK4a*^ in all adenocarcinoma cases. (**D**) Survival curves by methylation status of *RASSF1A* in all adenocarcinoma cases. The resulting curves were compared using log-rank test. M, methylated group; NM, not methylated group.

). The results of univariate and multivariate survival analysis for all variables are shown in Table 1a. Besides *p16*^*INK4a*^ methylation, T stage (*P*=0.0002), lymph node status (*P*<0.0001) and disease stage (*P*<0.0001) were correlated with poor prognosis on univariate analysis (Table 1a). On multivariate analysis, *p16*^*INK4a*^ methylation (RR=1.82, 95% CI=1.10–3.00, *P*=0.019), T stage (RR=1.87, 95% CI=1.20–2.92, *P*=0.006) and lymph node status (RR=4.60, 95% CI=3.00–7.05, *P*<0.0001) were independently associated with adverse prognosis. Since a previous study indicated that *RASSF1A* methylation was significantly related to poor prognosis in stage I adenocarcinoma (Tomizawa *et al*, 2002), we analysed our 105 cases of stage I adenocarcinoma (Table 1b). Of the variables, only *p16*^*INK4a*^ methylation was significantly related to unfavourable survival by univariate and multivariate analysis in this cohort (RR=2.57, 95% CI=1.15–5.76, *P*=0.022). There was no relationship between *RASSF1A* methylation (*P*=0.22) and survival in stage I adenocarcinoma or in all cases of adenocarcinomas (*P*=0.16) (Figure 1).

## DISCUSSION

Using newer molecular biology technologies, many studies for genetic abnormality of lung cancers have been investigated in cancer pathogenesis and for their effects on clinical outcome. Among the frequent genetic changes in lung cancer, abnormality of *p53* or *K-ras* has been extensively studied (Fukuyama *et al*, 1997; Mitsudomi *et al*, 2000) including its significant association with prognosis. Studies on the relationship between epigenetic changes and patient outcome are of more recent origin. In our analysis, only *p16*^*INK4a*^ methylation of the five genes that were frequently methylated in lung cancer was significantly correlated with poor prognosis in patients with lung adenocarcinoma. Furthermore, *p16*^*INK4a*^ methylation, along with lymph node status and T stage, was an independent prognostic factor by multivariate analysis. The p16^INK4a^ protein controls the transition from the G1 phase to the S phase in the cell cycle by inhibiting the phosphorylation of the retinoblastoma protein (Weinberg, 1995). Loss of *p16*^*INK4a*^ expression is frequently observed in lung cancers (Kratzke *et al*, 1996), and while inactivation may occur by other mechanisms such as point mutations or homozygous deletions, aberrant methylation is the most frequent method. Our results are consistent with other reports that loss of *p16*^*INK4a*^ expression was correlated with poor prognosis (Kratzke *et al*, 1996; Kawabuchi *et al*, 1999; Niklinski *et al*, 2001). In addition, Kim *et al* (2001) reported that *p16*^*INK4a*^ methylation was a risk factor predicting shorter survival after surgery. Reports with regard to *RASSF1A* are more contradictory (Burbee *et al*, 2001; Tomizawa *et al*, 2002; Endoh *et al*, 2003). We have previously reported an association between *RASSF1A* methylation and poor survival in resected Australian NSCLC cases (Burbee *et al*, 2001). In our present much larger study from Japanese cases, we were unable to demonstrate a relationship between *RASSF1A* methylation and survival despite analysis of the various subgroups. With regard to this issue, Tomizawa *et al* (2002) pointed out that *RASSF1A* methylation was correlated with poor prognosis in NSCLC patients at stage I. On the other hand, Endoh *et al* (2003) reported that there was no correlation. Some of these differences may be due to geographic, stage, subtype or other variables.

Our study represents the largest study correlating methylation and prognosis in lung cancer, and confirms and extends previous reports that p16^INK4a^ inactivation is a negative prognostic factor for NSCLC.
